# Endocrine-related adverse conditions in patients receiving immune checkpoint inhibition: an ESE clinical practice guideline

**DOI:** 10.1530/EJE-22-0689

**Published:** 2022-09-23

**Authors:** Eystein S Husebye, Frederik Castinetti, Sherwin Criseno, Giuseppe Curigliano, Brigitte Decallonne, Maria Fleseriu, Claire E Higham, Isabella Lupi, Stavroula A Paschou, Miklos Toth, Monique van der Kooij, Olaf M Dekkers

**Affiliations:** 1 Department of Clinical Science and K.G. Jebsen Center of Autoimmune Diseases, University of Bergen, Bergen, Norway; 2 Department of Medicine, Haukeland University Hospital, Bergen, Norway; 3 Aix Marseille Univ, INSERM U1251, Marseille Medical genetics, Department of Endocrinology, Assistance Publique-Hopitaux de Marseille, 13005 Marseille, France; 4 Department of Endocrinology, University Hospitals Birmingham NHS Foundation Trust, Birmingham, UK; 5 Department of Oncology and Hematology, University of Milan, European Institute of Oncology, IRCCS, Milan, Italy; 6 Department of Endocrinology, University Hospitals Leuven, Leuven, Belgium; 7 Pituitary Center, Department of Medicine and Neurological Surgery, Oregon Health & Science University, Portland, Oregon, USA; 8 Department of Endocrinology, Christie Hospital NHS Foundation Trust, University of Manchester, Manchester, UK; 9 Endocrine Unit, Pisa University Hospital, Pisa, Italy; 10 Endocrine Unit and Diabetes Centre, Department of Clinical Therapeutics, Alexandra Hospital, School of Medicine, National and Kapodistrian University of Athens, Athens, Greece; 11 Department of Internal Medicine and Oncology, ENETS Center of Excellence, Faculty of Medicine, Semmelweis University, Budapest, Hungary; 12 Department of Medical Oncology; 13 Department of Clinical Epidemiology, Leiden University Medical Center, Leiden, The Netherlands

## Abstract

Immune checkpoint inhibitors (ICI) have revolutionized cancer treatment but are associated with significant autoimmune endocrinopathies that pose both diagnostic and treatment challenges. The aim of this guideline is to provide clinicians with the best possible evidence-based recommendations for treatment and follow-up of patients with ICI-induced endocrine side-effects based on the Grading of Recommendations Assessment, Development, and Evaluation system. As these drugs have been used for a relatively short time, large systematic investigations are scarce. A systematic approach to diagnosis, treatment, and follow-up is needed, including baseline tests of endocrine function before each treatment cycle. We conclude that there is no clear evidence for the benefit of high-dose glucocorticoids to treat endocrine toxicities with the possible exceptions of severe thyroid eye disease and hypophysitis affecting the visual apparatus. With the exception of thyroiditis, most endocrine dysfunctions appear to be permanent regardless of ICI discontinuation. Thus, the development of endocrinopathies does not dictate a need to stop ICI treatment.

## Introduction

Over the past decade, immune checkpoint inhibitors (ICI) have revolutionized oncological treatments. These drugs are monoclonal antibodies targeting specific cell surface molecules (checkpoints) present on various cell types of the immune system that harness immune reactions. By releasing these endogenous immunological breaks, immune recognition and destruction of cancer cells can take place. In highly aggressive cancers such as malignant melanoma and lung cancer, ICI treatment has led to dramatically increased survival (1, 2, 3). However, these benefits come with the cost of autoimmune side-effects that affect a number of tissues, including the endocrine glands.

**Figure 1:**
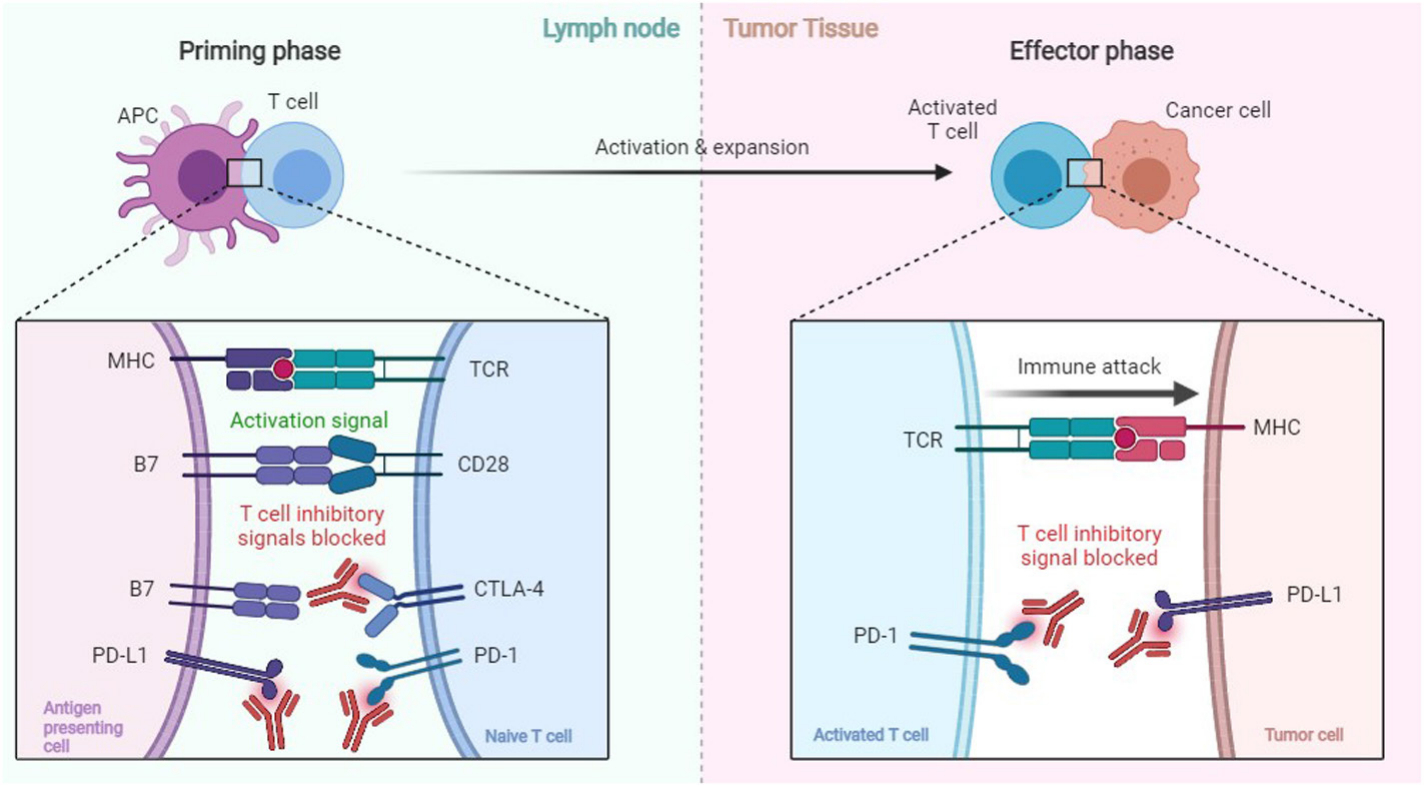
Mechanism of action of immune checkpoint inhibitors. Left: the priming phase of the T cell is shown. Antigen-presenting cells (APC) activate a naïve T cell by presenting a tumor antigen using their major histocompatibility complex (HMC), which is recognized by the T cell receptor (TCR). Possible inhibitory signals are blocked by anti-PD-L1, anti-PD-1, and anti-CTLA-4. Right: the activated T cell infiltrates the tumor tissue. There it recognizes the tumor antigen, which initiates an immune attack. Possible inhibitory signals are blocked by ant-PD-1 and anti-PD-L1.

At present, the two most frequently targeted immune checkpoints are the cytotoxic T-lymphocyte-associated antigen 4 (CTLA-4) and programmed cell death protein 1 (PD-1) (Fig. 1). CTLA-4 is constitutively expressed on the cell surface of regulatory T cells (Tregs), while its expression is inducible on conventional T-cells (4). CTLA-4 competes with CD28 for binding to CD80 or CD86 on antigen-presenting cells, thereby inhibiting immune activation (Fig. 1). PD-1 functions as a cell surface receptor belonging to the immunoglobulin superfamily and binds two ligands, programmed death-ligand 1 and 2 (PD-L1 and PD-L2). PD-L1 is expressed on cell surfaces of various tissues and cell types, including tumor cells. PD-L2 is mainly restricted to hemopoietic cells. In a significant proportion of cancer patients, monoclonal antibodies blocking CTLA-4, PD-1, and PD-L1 have durable anti-neoplastic effects (5).

The first ICI registered for human use was ipilimumab, an anti-CTLA-4 antibody, approved for metastatic melanoma in 2011 (6). Since then, several other monoclonal antibodies have been approved, by both the United States Food and Drug Administration (FDA) and the European Medicinal Agency (EMA), either as monotherapy or as combination therapy with other ICIs or conventional chemotherapy (7). Depending on tumor type, stage, and resectability, ICIs are used as either first-, second-, or third-line therapy or even in adjuvant or neo-adjuvant settings. Their indications are frequently related to various tumor-specific features, such as expression of PD-1/PD-L1, microsatellite instability, B-RAF protooncogene (BRAF) status (8), and tumor mutational burden (9).

At present, the CTLA-4 inhibitor ipilimumab; PD-1 inhibitors nivolumab, pembrolizumab, and cemiplimab; and PD-L1 inhibitors atezolizumab, avelumab, and durvalumab are FDA- and EMA-approved. The main fields of their usage are summarized in Supplementary Table 1 (see section on supplementary materials given at the end of this article). As of December 2021, these 7 antibodies have been approved in 18 cancer types for approximately 50 different indications. At present, one combination (ipilimumab and nivolumab) is approved for the treatment of melanoma.

The types of adverse events associated with immunotherapy are radically different from those with traditional cancer therapies (10, 11). Among the most frequent adverse events are colitis, pneumonitis, hepatitis, and cutaneous manifestations followed by endocrine disorders of the pituitary and thyroid glands (12). Fortunately, the majority of adverse events are mild (Common Terminology Criteria for Adverse Events (CTCAE) grades 1 and 2 in severity). Immune-related adverse events may result in chronic destruction and permanent loss of function of the affected organ. In most cases, adverse events develop in the first few weeks or months, but they can also develop at anytime during ICI therapy, even following the cessation of treatment (12). Whether patients with immune-related adverse events have better overall oncologic response rates to ICIs is still under intensive investigation. Recently published studies and systemic meta-analyses found that anti-PD-1 inhibitor-induced immune-related adverse events (including endocrine AEs) were strongly correlated with improved progression-free and overall survival in patients with non-small cell lung cancer (13), hepatocellular cancer (14), and other types of cancer (15).

Here we will focus on treatment and follow-up recommendations on ICI-induced endocrinopathies, as these areas are insufficiently covered by recently published guidelines (4, 16, 17, 18, 19, 20, 21).

### Diagnosis of endocrine side-effects

Symptoms and signs suggestive of endocrine adverse effects may appear briefly after initiation of ICI treatment, during the whole course of ICI treatment, and after ICI treatment is terminated. Signs and symptoms are diverse and relate to the endocrine organ affected (22). It can sometimes be difficult to differentiate the symptoms and signs of endocrinopathies from the effects of cancer itself or other treatments used in conjunction with ICI. For example, weakness, fatigue, increased sweating, tachycardia, weight change, polydipsia, nausea, vomiting, and abdominal pain are all associated with endocrine disturbance but are also common in patients with cancer undergoing treatments with cytotoxic drugs (10, 17, 23). Thus, biochemical surveillance of hormone levels is essential for a correct diagnosis, especially in patients with new or increasing signs and symptoms such as fatigue and weakness (24).

To date, at least six guidelines on endocrine complications of ICIs have been published by national and international endocrine and oncologic scientific societies (Table 1). Some variations and discordances between the guidelines exist related to recommendations on frequencies of endocrine testing, how to diagnose thyroid dysfunction, and adrenal insufficiency. Based on these guidelines, we suggest the following test regimens.

**Table 1:** Recommendations for diagnosis of endocrine complications in patients on immune checkpoint inhibitors according to various guidelines.

	SITC 2017 (78)	ASCO 2018 (10)	British Society for Endocrinology 2018 (acute setting) (19)	IDSC 2018 (18)	French Endocrine Society 2019 (17)	Japanese Endocrine Society 2019 (focus on management) (16)
Baseline	Clinical evaluation for symptoms and signs FBC, TSH, fT4, glucose, HbA1c, cortisol (8:00 h), ACTH (8:00 h)	Clinical evaluation for symptoms and signs TSH, fT4, glucose (fasting), electrolytes, cortisol (morning)	Clinical evaluation for symptoms and signs Random cortisol and ACTH FBC, TSH, fT4, glucose Prolactin, testosterone/estradiol, LH/FSH	TSH, FT4 for all Consider cortisol (8:00 h), ACTH (8:00 h) prior to anti-CTL4 therapy	Clinical evaluation for symptoms and signs Glucose (fasting, only with anti-PD-1/PD-L1), Na, TSH, fT4, cortisol (08:00 h) LH, FSH, testosterone (males), FSH (postmen. females) LH, FSH, estradiol in premen. Women with irregular menstrual cycle	n/a
Repeat of baseline tests	TSH, fT4, glucose, HbA1c before each cycle Cortisol (8:00 h), ACTH (8:00 h) should be considered (every month for 6 months, then every 3 months for 6 months then every 6 months for 1 year)	Repeat baseline evaluation every 4–6 weeks	n/a	TSH, FT4 at least monthly for the first 6 months, every 3 months during the next 6–12 months, every 6 months thereafterCortisol (8:00 h), +/− ACTH (8:00 h) for anti-CTL4 therapy at least monthly during the first 6 months, every 3 months during the next 6 months, every 12 months thereafter	Glucose (fasting, only with anti-PD-1/PD-L1), sodium, TSH, fT4, cortisol, testosterone in males at each appointment during the first 6 months and at every second appointment over the following 6 months, then on appearance of clinical signs	n/a
Additional Tests for:						
Hypothyroidism	Anti-TPO If levothyroxine started, TSH and fT4 in 6–8 weeks; Once a maintenance dose is identified, evaluation every 12 months	Monitor TSH every 6–8 weeks8 weeks while titrating hormone replacement to normal TSH. fT4 can be used in the short term (2 weeks) to ensure adequacy	If TSH > 10 mIU/L or TSH 4–10 mIU/L with low FT4 and/or symptoms, start levothyroxineIf not the above, repeat TFTs If TSH normal and fT4 below range, consider hypophysitis	If levothyroxine started, TSH every 4–6 weeks6 weeks while titrating hormone replacement. If no levothyroxine, close follow-up If TSH low or normal and fT4 below range, consider hypophysitis	Anti-TPO, if TSH: 5–10 mIU/L Thyroid ultrasound (+/−)Thyroid scintigraphy (+/−)During treatment with levothyroxine, monitoring with every 3 months	TSH, fT4, fT3 Anti-TPO, anti-TG Thyroid ultrasound
Hyperthyroidism	Anti-TPO TRAb (TSI) Thyroid scintigraphy (+/−)If no certain diagnosis, repeat TFTs every 2–3 weeks	Consider TRAb (TSI) if suspicion of Graves’ disease Close monitoring of thyroid function every 2–3 weeks to catch transition to hypothyroidism (due to destructive thyroiditis)	If persistent symptoms, consider autoimmune thyrotoxicosis If no symptoms, repeat TFTs with subsequent cycle	TFTs every 2–3 weeksTRAb (TSI) Thyroid scintigraphy	TRAb (TSI) Thyroid ultrasound (+/−)Thyroid scintigraphy (+/−)Clinical and hormonal monitoring for mild thyrotoxicosis	TSH, fT4, fT3 TRAb (TSI) Thyroid ultrasound
Adrenal insufficiency	Consider 1 μg ACTH stimulation test for secondary adrenal insufficiencyPrimary adrenal insufficiency is described as rare and no additional tests are recommended	Morning ACTH Consider ACTH stimulation test for indeterminate results If primary adrenal insufficiency (high ACTH, low cortisol) is found biochemically: adrenal CT	If primary adrenal insufficiency (normal or high ACTH), assess renin/aldosterone	Primary adrenal insufficiency is described as rare – no routine recommendations ACTH stimulation test may help Adrenal CT may help	ACTH measurement in patients with 08:00 h cortisol <18 μg/dLIf 8:00 h cortisol 5–18 μg/dL, Synacthen 250 μg testAnti-21 hydroxylase antibodies Adrenal CT, if primary adrenal insufficiency	Cortisol (morning), ACTH (morning), electrolytes, glucose ACTH stimulation test Renin or PRA Adrenal CT
Hypophysitis	Consider 1 μg ACTH stimulation testTestosterone (men), estradiol (women), FSH, LH Pituitary MRI	Consider LH, FSH and testosterone (males) or estrogen (premenopausal females) when fatigue, loss of libido and mood changes Consider pituitary MRI in patients with multiple endocrine abnormalities, new severe headaches or complaints of vision changes	Remainder of pituitary function, if not already measured (LH/FSH, estradiol/testosterone, PRL, IGF-1) If suspicion of hypopituitarism: pituitary MRI with contrast If significant polyuria/polydipsia, consider DI	Pituitary MRI Evaluate remainder of pituitary function Electrolytes Check vision	PRL Check for clinical signs of DI Pituitary MRI In patients with confirmed hypophysitis, clinical and hormonal monitoring (anterior pituitary hormone tests to check for new deficiencies and to adjust replacement therapy) at each appointment for 6 months, then at 3-monthly specialist consultations for 6 months and bi-annually thereafter	Evaluation for symptoms of hypopituitarism Pituitary hormones and hormones secreted by targeted organs Stimulation tests
Hyperglycemia	Blood or urine ketones Tests for antibodies (GADA, IAA, anti-islet cell, ZnT8A) Insulin and c peptide	If type 1 diabetes suspected: pH, urine ketones, GADA, IA2A, IAA Insulin and c peptide levels can assist	Monitor and manage for diabetic ketoacidosis or hyperglycemic hyperosmolar state Pancreatic antibodies (e.g. GADA) C-peptide	Autoantibodies (GADA, IAA, islet-cell ZnT8A) Insulin and c peptide	HbA1c GADA, IA2A, ZnT8A Lipase	Glucose, HbA1c Blood or urine ketones GADA C peptide
Hypoparathyroidism	n/a	n/a	n/a	n/a	n/a	Evaluation for symptoms of hypocalcemia PTH, Ca, P, Mg, 25OHD

25OHD, 25-hydroxy vitamin D; ACTH, adrenocorticotropic hormone; Ca, calcium; DI, diabetes insipidus; FBC, full blood count; FSH, follicle-stimulating hormone; fT3, free triiodothyronine; fT4, free thyroxine; GADA, glutamic acid decarboxylase autoantibodies; HBA1c, glycated hemoglobin; IAA, insulin autoantibodies; IA2A, tyrosine phosphatase-related antigen 2 autoantibodies; IGF-1, insulin-like growth factor 1; LH, luteinizing hormone; Mg, magnesium; n/a, non available, PRA, plasma renin activity; PRL,prolactin; PTH, parathyroid hormone; P, phosphate; TFTs, thyroid function tests; TG, thyroglobulin; TPO, thyroid peroxidase; TRAb, TSH receptor antibodies; TSH, thyroid-stimulating hormone; T3, triiodothyronine; ZnT8A, zinc transporter autoantibodies.

*Baseline minimum tests*: Morning (8:00–9:00 h) thyroid-stimulating hormone (TSH), free thyroxine (fT4), cortisol (with attention to any recent or current glucocorticoid treatment), glucose, and electrolytes (Na, K, Ca). These tests should be repeated every 4–6 weeks, preferably before each treatment cycle starts.

*Comprehensive baseline assessment*: Morning (8:00–9:00 h) adrenocorticotropic hormone (ACTH), luteinization hormone (LH), follicle-stimulating hormone (FSH), estradiol (pre-menopausal females), testosterone (males), prolactin, and glycated hemoglobin (HbA1c).

The baseline minimum tests should be performed in all patients and the comprehensive tests should be used when there is an increased risk for hypophysitis, for instance, when ipilimumab is used alone or in combination with a PD-1 inhibitor. For the diagnosis of specific endocrinopathies, additional tests may be indicated as suggested in each sub-section below.

## Methods

### Guideline working group

This guideline was developed on behalf of The European Society of Endocrinology (ESE), the chairs of the working group, Eystein S. Husebye and Olaf M. Dekkers were appointed by the ESE Clinical Committee. Olaf M. Dekkers served as methodological expert, Maria Fleseriu as representative of The Endocrine Society, USA, Sherwin Criseno as ESE Nurses Representative, Stavroula A. Paschou as ESE Young Endocrinologists and Scientists representative, and Guiseppe Curigliano as The European Society for Medical Oncology representative. The other members were suggested by the chairs and approved by the Clinical Committee of ESE, including Claire Higham, Brigitte Decallonne, Fredric Castinetti, Miklos Toth, and Isabella Lupi. Monique van der Kooij joined the guideline working group for methodology support. Due to COVID restrictions, all three working group meetings were held virtually and further communication was done by email. Consensus was reached upon discussion; minority positions were taken into account in the rationale behind recommendations. Prior to the process, all participants completed conflict of interest forms.

### Target groups

This guideline was developed for health care professionals who may see patients with endocrine-related adverse conditions during or after ICI treatment and who seek guidance for the treatment and follow-up. General practitioners, nurses, and patients might also find the guideline useful. The guideline can serve as a source document for the preparation of patient information leaflets and educational materials.

### Aims

The purpose of this guideline is to provide clinicians with practical guidance on the management of patients with ICI-related endocrine conditions. In clinical practice, both the recommendations and the clinical judgment of the treating physician should be taken into account. Recommendations are not meant to replace clinical acumen and may need adaptation to local circumstances.

### Summary of methods used for guideline development

The methods used for establishing the guideline have been described in detail previously (25). In short, we used Grading of Recommendations, Assessment, Development, and Evaluation (GRADE) as a methodological basis. The first step was to define the clinical questions (see below) followed by a systematic literature search. We rated the quality of the evidence and estimated an average effect for specific outcomes where possible. The quality of the evidence behind the recommendations was classified as very low (*+000*), low (*++00*), moderate (*+++0*), or strong (*++++*). Not all recommendations were formally graded (see below).

For the recommendations, we considered the quality of the evidence, the balance of desirable and undesirable outcomes, and individual values and preferences (patient preferences, goals for health, costs, management inconvenience, feasibility of implementation) (25, 26). The recommendations are worded as ‘recommend’ (strong recommendation) or ‘suggest’ (weak recommendation). The meaning of a strong recommendation is that all reasonably informed persons (clinicians, politicians, and patients) would want the management in accordance with the recommendation. For a weak recommendation, most persons would still act in accordance with the guideline, but a substantial number would not. Formal evidence syntheses were performed and graded only for recommendations addressing our initial clinical questions (see below). Other recommendations were based on good clinical practice and experience of the panelists and are not formally graded; this is acknowledged in the document. Recommendations were derived from a majority consensus of the guideline development committee. Potential disagreements are acknowledged. All recommendations are accompanied by an explanation.

### Clinical questions, eligibility criteria, and definition of endpoints

At the start of this guideline process, the committee members formulated clinical questions regarding treatment and follow-up of ICI-related endocrine conditions. The clinical questions that formed the basis of the systematic literature search and review are summarized in Supplementary Table 2.

Cohort studies assessing the prevalence and treatment of ICI-related endocrine adverse events in patients with cancer who received ICI were eligible for inclusion. Eligible studies included adult patients (>18 years old). All cancer subtypes were eligible, for which treatment with ICI was approved by the FDA and EMA. ICI treatments included: anti-CTLA-4 (ipilimumab), anti-PD-1 (nivolumab, pembrolizumab, cemiplimab), anti-PD-L1 (atezolizumab, avelumab, durvalumab), and combination treatment of ipilimumab and nivolumab. Combination treatment with ICI and chemotherapy or ICI with targeted therapy was not considered. Only studies reporting on ten or more patients were considered eligible in order to avoid selection bias. Eligible studies were restricted to languages familiar to the authors.

The following endocrine adverse conditions related to ICI treatment were considered: hypophysitis, thyroid disorders (Graves’ disease, thyroiditis, hypothyroidism, thyroid eye disease), adrenal insufficiency, diabetes mellitus, hypoparathyroidism, and polyendocrine syndromes. Interventions were divided into three different categories: (i) pharmacological glucocorticoid treatment for management of the endocrinopathy (prednisolone, dexamethasone), (ii) hormone replacement therapy (hydrocortisone, levothyroxine, insulin, testosterone/estrogen, desmopressin) or anti-thyroid drugs, and (iii) dose modification, holding, or discontinuation of ICI treatment. The control was defined as no intervention or best supportive care. In addition, studies without interventions were considered for information on the natural course of the endocrinopathy. Outcome measures were recovery from the adverse event, ongoing or permanent organ dysfunction, hospitalization, and adverse event-related mortality. Details of included studies are shown in Supplementary Table 3, the GRADE assessment in Supplementary Table 4.

### Description of search and selection of literature

In October 2020, PubMed, EMBASE, EMCARE, Web of Science, and COCHRANE Library were searched to identify potentially relevant studies, published since the introduction of ICIs in clinical practice in 2010. All studies obtained from this search strategy were entered into reference manager software (EndNote X9, Clarivate Analytics, Philadelphia, PA) and screened for title and abstract. Potentially relevant studies were retrieved for detailed assessment.

### Review process and endorsement by other societies

A draft of the guideline was reviewed by four experts in the field (see ‘Acknowledgments’ section) and was distributed to all ESE members for comments. In addition, the following societies and networks were asked to review the guidelines; Endocrine Society, International Endocrine Society, and European Society for Medical Oncology. All comments and suggestions were then discussed and implemented as thought appropriate by the panel.

## General recommendations

Based on a group consensus, we arrived at the following general recommendations:

**R 1.1** - We recommend that a controlled endocrinopathy is not a contraindication for initiation and/or continuation of ICI therapy (++00).

**Rationale** ***:*** The oncologic benefit of ICI treatment outweighs the risk related to ICI-induced endocrinopathies, in almost all instances. Rare cases (adrenal crisis, severe thyrotoxicosis) may need endocrine control first, but this can be done within days to weeks and should not stop or delay treatment with ICI significantly. Several studies have reported on the safety and efficacy of ICI treatment in patients with pre-existing autoimmune diseases. A recent cohort study including 143 metastatic melanoma patients with a pre-existing endocrine autoimmune disease (mostly thyroid) showed that these patients neither develop more endocrine-related CTCAE grades 3–4 adverse events following ICI nor did they have a lower response rate or survival (27). Data from this study correspond with other smaller studies (28, 29).

**R.1.2** - We suggest a discussion between oncology and endocrinology with regard to the possible interruption of ICI in the case of severe thyroid eye disease or hypophysitis with compression of the optic chiasm or the optic nerve (30); for other endocrine abnormalities, there is no need to interrupt or stop ICI treatment (++00).

**Rationale** ***:*** There is no documented effect of stopping ICI treatment on the recovery of endocrine function.

**R.1.3** - For patients with sustained and/or multiple endocrine deficiencies and a good oncological prognosis, an endocrinologist should be involved in the ongoing endocrine care, with the exclusion of isolated hypothyroidism which can be managed by other medical professionals such as primary care or the treating oncologist (good clinical practice).

**Rationale** *:* Endocrine conditions listed other than primary hypothyroidism are usually cared for by an endocrinologist. They are chronic lifelong conditions with long-term complications if not treated properly.

**R.1.4** - We recommend that patients with ICI-induced endocrine dysfunctions are provided with adequate education and training on effectively managing their conditions including provision of appropriate verbal and written instruction on how to adjust their medications during periods of illness.

## Recommendations on specific endocrinopathies

### Pituitary

The overall incidence of hypophysitis is up to 17% in patients treated with ICI; with a female: male ratio of 1:4. The mean age at onset is approximately 60 years and mean time to onset is 10.5 weeks for CTLA-4 inhibitors and 27 weeks for PD-1/PD-L1 inhibitors (31). The prevalence of hypophysitis depends on the type and dose of ICI. Out of 276 patients with hypophysitis described in the literature between 2003 and 2019, 70% were secondary to CTLA-4 blockade, 23% to PD-1 blockade, 2% to PD-L1 blockade, and 3.9% to combination therapy (CTLA-4 and PD-1) (31, 32). Between 1.8% and 3.3% developed hypophysitis on low doses of ipilimumab (<3 mg/kg) vs 4.9–17% of patients on doses >3 mg/kg (32).

ICI-induced hypophysitis has several distinctive features compared with autoimmune (primary) hypophysitis. The former is more common in males, presents at an older age, and almost always with symptoms and signs of hypocortisolism, whereas manifestations related to pituitary enlargement are overall moderate (33, 34, 35, 36).

Pituitary antibodies assayed by indirect immunofluorescence on human pituitary sections were found in 15 out of 17 (88.2%) and 4 out of 5 (80%) patients treated with anti-PD1 or anti-CTLA-4, respectively (37). The pituitary autoantigen(s) has not been identified.

Clinical presentations of hypophysitis vary depending on ICI type and dose. In anti-CTLA-4-induced hypophysitis, ACTH deficiency is most common (95%) and is often accompanied by other pituitary insufficiencies (31). Secondary hypothyroidism was described in 85% and secondary hypogonadism in 75%. The true prevalence of GH deficiency is unknown due to lack of routine dynamic testing of the GH axis, based on the rationale that GH treatment would not be indicated in the presence of active cancer. MRI abnormalities are seen in 81% of cases due to anti-CTLA-4 treatment and in 18% of patients with a hypophysitis who were treated with anti-PD-1/anti-PD-L1, typically with an initial enlargement of the pituitary, which returns to normal within weeks (31). Most patients treated with an anti-CTLA-4 develop hypophysitis within several weeks after treatment initiation (mean time to onset 10.5 ± 4.8 weeks). Anti-PD-1-induced hypophysitis often presents with isolated ACTH deficiency (97%). Hyponatremia tends to be one of the most common presenting findings (63%) and MRI abnormalities are seen in only 18% of cases. Anti-PD-1-induced hypophysitis can develop several months to over a year after treatment initiation (31). Involvement of the posterior pituitary with central diabetes insipidus (DI) is overall rare, only described in 3% of anti-PD-1- and anti-CTLA-4-induced hypophysitis (31, 38, 39).

#### Diagnostic considerations

Prior to evaluating pituitary function, any concomitant use of drugs that may interfere with pituitary function or with hormone assays should be identified. Screening for pituitary dysfunction should be done as per current guidelines (40). Notably, glucocorticoids at high doses cause ACTH suppression and can also suppress TSH, gonadotropins, GH, and vasopressin secretion. Opiates, often used in these patients to alleviate pain, suppress ACTH and gonadotropin secretion (41). Diabetes insipidus (DI) is rare, but monitoring is valuable, especially after starting glucocorticoids and opiates (19, 23). Pituitary MRI can help confirm the diagnosis of hypophysitis and exclude other causes of pituitary failure such as metastatic disease. The latter is characterized by intense heterogenous pattern of enchancement and extension into the cavernous sinus (42). A normal scan does not exclude hypophysitis (43).

#### Summary of evidence

Seven observational studies have been published studying pituitary insufficiency in patients after ICI treatment (34, 35, 39, 44, 45, 46, 47). Often detailed information on the type or severity of the pituitary insufficiency was available. Recovery of ACTH deficiency is extremely rare, although it had been reported after high-dose glucocorticoids. No further evidence exists for the benefit of high-dose glucocorticoids to reverse pituitary insufficiency. Recovery of TSH-deficiency and gonadotroph deficiency is more often reported. See Supplementary Tables 3 and 4 for details.

#### Recommendations for treatment and follow-up

**R 2.1** - We recommend the use of high-dose glucocorticoids in case of an acutely unwell patient ICI-treated with suspected adrenal crisis, more specifically 100 mg hydrocortisone intravenously or intramuscularly followed by 50 mg every 6 h and fluid resuscitation (48) (good clinical practice).

**Rationale** ***:*** A suspected adrenal crisis should be treated after securing a blood sample for cortisol and preferably also ACTH testing. Do not delay treatment for adrenal crisis while waiting for these results as this can be fatal.

**R 2.2** - We recommend against the use of high-dose glucocorticoids for treatment of hypophysitis, with exception of optic chiasm compression or other severe compressive symptoms. (++00)

**Rationale:**There is currently no evidence for the efficacy of high-dose glucocorticoid treatment, and there may actually be an increased mortality risk with this approach (39). For pituitary enlargement/compression symptoms including headache and visual disturbances, however, high-dose glucocorticoids have been proven useful (34, 39, 49). Conversely, one study reported that patients treated with high-dose glucocorticoids had reduced survival (39). It is unclear whether this was due to effects of high-dose glucocorticoids or if these patients had a worse oncologic prognosis at baseline. In the acute phase of ICI-induced hypophysitis without compressive symptoms, treatment with high-dose glucocorticoids is not recommended, as resolution of hypopituitarism is not superior compared to patients receiving glucocorticoid replacement therapy (34, 35). There is also a risk of inducing secondary adrenal insufficiency if high-dose glucocorticoids are used over longer periods of time. We recommend to reserve high-dose glucocorticoids for the rare cases with compromised vision (large volume hypophysitis) and severe untreatable headache (case reports, also from non-ICI-treated patients).

**R 2.3** - We recommend standard glucocorticoid replacement therapy for patients with hypophysitis and confirmed ACTH deficiency. Most patients are managed well on a daily dose of 15–25 mg hydrocortisone split into two or three doses (40) (good clinical practice).

**Rationale:**There is little evidence for spontaneous recovery of pituitary function after ICI is stopped (31, 46). Thus, regular oral replacement therapy should be used, preferably a total daily dose of hydrocortisone of 15–25 mg given twice or trice daily (or cortisone acetate 20–30 mg daily) (40). When available, the extended release formulation of hydrocortisone, Plenadren® can be used at a dose of 20 mg once daily. Prednisolone at 3–4 mg daily is also an alternative (50), but dexamethasone has no place in replacement therapy as there is a high risk of Cushingoid side-effects and suppression of the hypothalamic–pituitary–adrenal axis (HPA). All patients should appropriate and regular training on stress doing (‘Sick Day Rules’) and be equipped with hydrocortisone injection kit and steroid emergency card (51). Mineralocorticoids should not be given in secondary adrenal insufficiency.

**R 2.4** - We recommend against stopping high-dose glucocorticoid replacement abruptly without formal assessment of pituitary–adrenal function, even if ICI treatment was stopped (good clinical practice).

**Rationale:**Use of doses equivalent to 7.5 mg prednisolone or higher for more than 3 weeks can lead to secondary adrenal insufficiency. Thus, at least a medication fasting morning cortisol (plus ACTH) and if needed a 250 µg Synacthen® test should be considered if glucocorticoids are considered to be stopped. The decision to taper the glucocorticoid dose and assess the HPA axis will depend on the prognosis of the patient. In patients with confirmed adrenal insufficiency on glucocorticoid replacement therapy, there is no indication for routine assessment of cortisol levels.

**R 2.5** - We recommend thyroid hormone replacement therapy in patients with hypophysitis who have decreased or low-normal fT4 (good clinical practice).

**Rationale:**As levothyroxine treatment can precipitate an adrenal crisis in a patient with untreated adrenal insufficiency, adrenal insufficiency should be excluded and glucocorticoids initiated when needed, prior to the start of levothyroxine. TSH cannot be used to monitor dosing in central hypothyroidism. It is therefore common to give a replacement dose that puts fT4 in the upper part of the reference range (52). The half-life of levothyroxine is about 1 week. Thus, allow 5–6 weeks before a dose change is assessed. We suggest to start with 1.0 µg/kg/d (52). In cardiovascularly compromised or elderly patients, a lower daily starting dose (e.g. 25–50 µg/d) can be considered. Recovery of the TSH axis may occur, usually within months following hypophysitis secondary to ICI (34, 35).

**R 2.6** - We suggest considering tapering and stopping levothyroxine periodically in patients with low-dose replacement to assess whether thyroid function has recovered (+000).

**Rationale** ***:*** Recovery of thyroid axis has been reported, this may indicate a resolution of central hypothyroidism, recovery of non-thyroidal illness or because other medications affected thyroid function tests (e.g. heparin, glucocorticoids, opiates, amiodarone, and iodine-based contrast agents) (34, 35, 39, 53).

**R 2.7** - We suggest not to directly correct gonadal dysfunction in the setting of a poor oncologic prognosis (good clinical practice).

**Rationale:**Suppression of the gonadotrophins axis by illness and treatment is common and should preferably be resolved before sex steroid replacement is considered. Furthermore, the age of the patient and oncologic prognosis must be taken into consideration. Since there are reports of reversibility of secondary hypogonadism, sex steroid replacement – when initiated – should be short term and evaluated again before long-term treatment is considered.

**R 2.8** - We suggest replacing gonadal hormones, with testosterone in men and estrogen and progestagen in women of reproductive age, in the setting of hypophysitis with persistent and confirmed hypogonadotropic hypogonadism (good clinical practice).

**Rationale:**For women, sex steroid replacement during reproductive age is important for well-being and to prevent complications such as osteoporotic fractures and cardiovascular disease. It is common to continue replacement to the age of normal menopause around 50 years of age. Consider transdermal estrogen administration to minimize thromboembolic risk. For men, testosterone is important for well-being, libido, bone health, muscle strength, and to avoid anemia. In the absence of any contraindications (for example hormone-sensitive cancers; prostate, ovarian, breast) or increased risk of acute thromboembolic complications, a decision with regards to gonadal replacement should be guided by the patient’s age, symptomatology, cancer type, and prognosis. Osteoporosis should be treated with calcium and vitamin D supplementation and bone-directed agents, if sex steroid replacement is not feasible.

**R 2.9** - We recommend against treating GH deficiency in patients with hypophysitis in the presence of an active malignancy.

**Rationale:**Although GH deficiency in the long term can affect morbidity, GH replacement therapy during malignancy, as in patients with ICI-induced hypophysitis, is contraindicated (40). The risk of secondary malignancies is increased in childhood-onset but not in adult-onset cancer survivors (54). As of now, there are no case studies to assess the effects of GH in the setting of ICI-hypophysitis.

**R 2.10** - We recommend that desmopressin is given to patients with hypophysitis and confirmed DI (good clinical practice).

**Rationale**: Desmopressin is needed to maintain normal osmolality and sodium concentration. Other causes of sodium imbalances should be considered. Aldosterone deficiency causes urinary sodium loss and cortisol deficiency impairs free renal water clearance. These conditions may mask the presence of central DI and must be corrected before testing for vasopressin deficiency. In a patient with polyuria (defined as urine output of more than 50 mL/kg/24 h), after exclusion of adrenal insufficiency and diabetes mellitus, serum and urine osmolarity are measured. Other causes, such as renal dysfunction, hypokalemia, or hypercalcemia, and the effects of diuretics should also be considered. In the presence of high serum osmolarity (above 295 mOsmol/L), a urine osmolality/serum osmolality ratio of two excludes the diagnosis of DI (40). Milder cases of hypotonic polyuria should be further evaluated with arginine-stimulated co-peptin or water deprivation tests. If DI is confirmed, vasopressin therapy is administered (sublingual, intranasal, or oral), carefully monitoring sodium and fluid balance (fluid intake and urine output) over 24 h. Overtreatment can lead to hyponatremia.

**R 2.11** - We recommend that patients with pituitary hormone deficiencies, more particularly ACTH and vasopressin deficiencies, are provided with appropriate and regular training on managing their glucocorticoid and/or desmopressin treatment during period of illness (‘Sick Day Rules’) (good clinical practice).

**Rationale**: Adrenal crises and poorly controlled DI can be avoided and/or alleviated by early treatment started either by the patient or a relative who has received education in these conditions. Thus, self-management should include training on stress dosing (‘Sick Day Rules’), self-administration of hydrocortisone injection in the event of an emergency and provision of appropriate resources (e.g. Steroid Emergency Card, identification bracelet/necklace, care plan outlining the management of adrenal crisis and peri-operative glucocorticoid requirement) to enable accurate communication of their conditions and treatment with other healthcare professionals. Consider referral to a patient support group (if available) for ongoing peer-support.

### Thyroid

Thyroid dysfunction represents the most common ICI-related endocrine adverse event. Anti-PD-1/PD-L1 treatment carries a higher risk than anti-CTLA4 treatment, with the highest risk for combined treatment (55). Most cases are asymptomatic or mild (grade I or II). Hypothyroidism is most frequent with an estimated incidence of 2.5–3.8% (anti-CTLA4), 3.9–8.5% (anti-PD1/PDL1), and 10.2–16.4% (combination treatment), typically occurring at a median time of 8–12 weeks post ICI initiation (56). In a minority of cases, especially in patients treated with anti-CTLA4, central hypothyroidism can develop (TSH deficiency), mostly along with ACTH deficiency which should not be missed and always be treated prior to the initiation of thyroid hormone replacement (see above). Thyrotoxicosis, typically preceding hypothyroidism, has a lower incidence estimated at 0.2–5.2% (anti-CTLA4), 0.6–3.7% (anti-PD1/PDL1), and 8.0–11.1% (combination treatment) (56). The incidence is believed to be underreported, as thyrotoxicosis is typically mild and transient and therefore at risk to remain undetected. Thyrotoxicosis has a rapid onset, typically 4–6 weeks post ICI initiation, and lasts approximately 6 weeks (57). The time to onset of thyrotoxicosis is shorter in case of ICI combination treatment. Rare cases of Graves’ hyperthyroidism and thyroid eye disease have been described (58, 59).

Most studies describe two common patterns of ICI-related thyroid dysfunction: thyrotoxicosis followed by hypothyroidism or isolated hypothyroidism. Hypothyroidism is often permanent, especially when overt. From the currently available studies, the proportion of transient vs permanent hypothyroidism cannot be defined. The risk for thyroid dysfunction seems not to be dependent on the cancer subtype, ICI dosage (60), or age. In contrast to hypophysitis, which is more common in males, thyroid dysfunction seems to occur more frequently in females (61). The broad variation in incidence of thyroid dysfunction mainly depends on the definition of thyroid dysfunction and the frequency of thyroid function testing. Other factors which could potentially affect or trigger thyroid dysfunction, such as iodine-based contrast agents, high-dose glucocorticoids for other indications, and non-thyroidal illness, are usually not taken into account. Patients with known hypothyroidism may need higher doses of levothyroxine after ICI initiation. Patients with increased ^18^F-deoxyglucose uptake in the thyroid (62, 63, 64), high TSH, and thyroid autoantibodies might be at higher risk (63, 65, 66), as well as patients with a high BMI (67). Several studies observed an association between ICI-induced thyroid dysfunction and overall survival, suggesting that the occurrence of thyroid dysfunction could represent a surrogate marker for anti-tumor response (65, 68, 69, 70).

The underlying pathophysiology is considered to be an immune-mediated acute inflammation followed by destruction of the thyroid gland, typically with hypovascularity on ultrasonography, low uptake on thyroid scintigraphy, and absence of TSH receptor-antibodies. The high expression of PD-L1/PD-L2 on thyrocytes could explain the high susceptibility (62). TPO-antibodies are variably positive (65, 71). A substantial proportion of patients could have an underlying latent chronic autoimmune thyroiditis, becoming overt under ICI treatment, rather than a *de novo* thyroiditis. ICI-induced thyroiditis also differs from tyrosine kinase-induced thyroid dysfunction, thought to be secondary to decreased thyroid perfusion (72), and from thyroid dysfunction after immune reconstitution (typically following treatment with alemtuzumab) (73). One study suggested an increased risk for ICI-induced thyroid dysfunction after prior treatment with tyrosine kinase inhibitors (74).

A recent review of ICI-associated neuro-ophthalmologic complications reported a total of 109 cases with enough details for analysis. The incidence of neuro-ophthalmologic complications is estimated at 0.46%. Thyroid eye disease was the fourth most common cause of ICI-associated neuro-ophthalmologic disorders (12% of the cases), following myasthenia gravis (45%), orbital myositis (14%), and optic neuritis (13%). Six out of ten cases were associated with Graves’ disease. Thyroid eye disease developed typically 2–4 months following initiation of ICI. Peroral or i.v. high-dose glucocorticoids were used in nine out of ten patients. ICI was held or terminated in the majority, while it was continued in three patients. Improvement or resolution was reported in all but one patient, even in two out of three patients with continued administration of ICI (75). The very low number of reported patients with ICI-associated thyroid eye disease does not permit formulation of recommendations regarding the continuation of ICIs (75, 76, 77).

#### Diagnostic considerations

As thyroid dysfunction is mild in the majority of cases, diagnosis relies on the combined measurement of TSH and fT4, also allowing the detection of central hypothyroidism. When thyroid dysfunction is overt, symptoms and signs should not be confounded with those related to the underlying malignant disorder or other side-effects of ICI treatment. Furthermore, thyroid tests can be influenced by other factors, such as drugs, non-thyroidal illness, and iodine-based contrast agents. All guidelines advise screening of thyroid function prior to the initiation of ICI, but it is generally not recommended to screen for TPO-antibodies (10, 16, 17, 18, 19, 78). The role of baseline TSH as a predictive marker for ICI-related thyroid dysfunction remains to be determined (79). Only in cases of overt thyroid dysfunction or a history of severe thyroid eye disease, there is a need to discuss the initiation of ICI treatment with a multidisciplinary team, which should include an endocrinologist.

During ICI treatment, it is generally advised to monitor TSH and fT4 prior to every ICI treatment cycle during the first 6 months, after which the interval can be prolonged to every 2–3 months for 6 months and every 6 months thereafter. In case of discrepant test results or non-concordance between clinical and biochemical findings, assay interference should be suspected (80). In case of thyrotoxicosis, guidelines generally recommend TSH receptor-antibody testing. In case of hypothyroidism, TPO-antibody testing is recommended as it indicates autoimmune etiology. Most guidelines do not recommend thyroglobulin antibody testing. Since cases of Graves’ disease have been described without increased levels of TSH receptor antibodies (58), fT3 testing, thyroid scintigraphy, and ultrasonography are useful for differentiation in cases of severe or prolonged thyrotoxicosis.

#### Treatment considerations

Most cases of thyrotoxicosis can be treated with beta-blockers for symptomatic relief. Graves’ disease requires treatment with anti-thyroid drugs. Primary hypothyroidism is treated with levothyroxine aiming at normalisation of TSH values.

#### Summary of evidence

Eight studies reporting on levothyroxine replacement therapy could be included in our systematic review (57, 62, 65, 68, 69, 81, 82, 83). In a substantial number of patients, thyroid dysfunction was reported, ranging from 14 to 33%. See Supplementary Tables 3 and 4 for details. A presentation with thyrotoxicosis first or hypothyroidism was both often reported. In some studies, all patients required levothyroxine replacement, while in other studies the percentages varied between 20 and 84%, especially in patients with mild subclinical hypothyroidism. Recovery after thyrotoxicosis was reported in up to 57%; some patients that did recover received high-dose glucocorticoids for other immune-related adverse events (57). However, most patients with hyperthyroidism progressed to hypothyroidism. Recovery from hypothyroidism was only rarely reported.

A retrospective analysis of high-dose glucocorticoid treatment of ICI-related thyroid disorders (15 patients treated vs 38 patients not treated with high-dose glucocorticoids) concluded that glucocorticoids did not prevent or reduce ICI-induced thyroid damage. There was no difference in maintenance dose of levothyroxine between patients receiving high-dose glucocorticoids (1.5 µg/kg/d, range 0.4–2.3) and those who did not (1.3 µg/kg/d, range 0.3–2.5), nor in time to onset of thyrotoxicosis, time to conversion to hypothyroidism, or time to onset of hypothyroidism (79). However, most studies did not apply high-dose glucocorticoid therapy. Prospective randomized studies are needed in order to evaluate whether high-dose glucocorticoids should be used in selected subgroups of patients with ICI-induced thyroid dysfunction to improve the outcome.

#### Recommendations for treatment and follow-up

**R 3.1** - We recommend against the use of high-dose glucocorticoids for the treatment of thyroiditis; except in severe thyrotoxicosis and severe thyroid eye disease (+000).

**Rationale:**There is no evidence for treatment efficacy of high-dose glucocorticoid therapy. Moreover, such treatment can induce secondary adrenal insufficiency if doses of prednisolone > 7.5 mg per day are given for longer than 2–3 weeks. In cases of severe thyrotoxicosis, high-dose glucocorticoids reduce T4 to T3 conversion. Cases of ICI-related thyroid eye disease are extremely rare but can occur even in the absence of increased TSH receptor-antibodies and should be referred to an endocrinologist and ophthalmologist for shared decision regarding treatment with high-dose glucocorticoids.

**R 3.2** - We suggest that cases of clinically and biochemically mild thyrotoxicosis or hypothyroidism can be monitored without treatment (good clinical practice).

**Rationale:**Most cases of thyroid dysfunction are mild. Monitor TSH and fT4 every 3–6 weeks in the early phase; the frequency can be lowered at later stages (depending on biochemical levels and trends) (84).

**R 3.3** - We recommend using beta-blockers in symptomatic thyrotoxicosis (good clinical practice).

**Rationale:**Short-term treatment with a non-selective beta-blocker (e.g. propranolol) can be initiated to resolve symptoms of thyrotoxicosis. As most cases are caused by thyroiditis, anti-thyroid drugs are not indicated, except in rare cases of Graves’ hyperthyroidism, which should be referred to an endocrinologist for shared care. Radioactive iodine and thyroid surgery are not recommended as first-line treatment of ICI-related thyrotoxicosis.

**R 3.4** - We suggest replacement with levothyroxine in primary hypothyroidism in cases with fT4 below the lower reference limit and TSH > 10 mIU/L. (++00)

**Rationale:**Symptoms and signs of hypothyroidism are difficult to evaluate in an oncological setting, but in cases of overt hypothyroidism, we recommend replacement as there is a significant chance of clinical improvement. A starting dose of 1.0 µg/kg/d (25 µg/d in elderly or in patients with cardiovascular comorbidity) is appropriate. Regular monitoring of thyroid hormones (TSH and fT4) is needed in order to titrate levothyroxine dosage at intervals of at least 6 weeks. In patients with low-dose replacement, we suggest considering tapering and stopping levothyroxine periodically to assess whether thyroid function has recovered (+000)

**R 3.5** - We recommend against routine replacement with levothyroxine of ICI-treated patients with a TSH between 4–10 mIU/L and a normal fT4 (+000).

**Rationale:**The decision depends on the level and trend of fT4 and symptoms. Regular monitoring of thyroid tests (TSH and fT4) and timely balancing of the benefit/risk ratio of initiating levothyroxine replacement are needed.

**R 3.6** - In case of ICI cessation, ongoing monitoring of thyroid function (TSH and fT4) is still indicated, albeit less frequently (e.g. every 6–12 months and in case of symptoms and signs of thyroid dysfunction).

**Rationale:**Thyroid dysfunction can develop at a later stage and symptoms are usually non-specific. Ongoing biochemical testing is therefore needed for at least two years (85).

### Pancreatic islets

The overall incidence of ICI-induced diabetes mellitus (DM) ranges from 0.9 to 2%. There is a female to male ratio ranging from 1:1.2 to 1:9, and a mean age at onset of over 60 years (86, 87, 88). Diabetic ketoacidosis (DKA) at diagnosis is very frequent and seen in up to 70% of cases. Up to 76% of those developing DM received anti-PD-1, 8% anti-PD-L1, while only 4% received anti-CTLA-4 (89, 90). ICI-induced DM develops rapidly, often with the presence of autoantibodies and HLA genotypes that are associated with type 1 DM. Indeed, in most cases, an immune-mediated destruction of the pancreatic islets takes place. Type 2 DM or glucocorticoid-induced DM should be always considered in oncological patients.

#### Diagnostic considerations

When hyperglycemia is present, urine ketone testing and blood pH measurement are recommended for early diagnosis of diabetic ketoacidosis (DKA). Use of SGLT2-inhibitors is an additional risk factor for DKA (91). HbA1c is not a good screening parameter due to the typically acute onset of hyperglycemia in ICI-induced DM, although it can reveal previous undiagnosed DM. Autoantibodies against glutamic acid decarboxylase (GAD), islet antigen-2 (IA2), insulin (IAA), and the zinc transporter ZnT8 are highly specific for autoimmune DM and are also reported in cases of ICI-induced DM. Insulin and C-peptide levels measurement can assist in the evaluation of endogenous insulin secretion.

#### Summary of evidence

Two studies were included in our systematic review, both showing that insulin treatment is mandatory for patients with DM following ICI treatment with (almost) no chance of subsequent insulin withdrawal (87, 92). The reported diabetes incidences were 0.8 and 2.3% following ICI treatment with either anti-PD-1 or anti-CTLA-4 monotherapy. In total, 29 patients with ICI-induced DM were reported. DM remission and insulin withdrawal were noted after ICI discontinuation in one single patient (92). See Supplementary Tables for details.

#### Recommendations for diagnosis and treatment

**R 4.1** - We recommend that patients presenting with DKA should be managed with i.v. insulin and fluid resuscitation according to current guidelines (93) (good clinical practice).

**Rationale:**ICI-induced DM typically develops rapidly and patients are prone to developing DKA if not treated aggressively. Hospitalization for treatment with i.v. insulin administration and hydration is most often necessary.

**R 4.2** - We recommend against the use of high-dose glucocorticoids for treatment of ICI-induced DM (+000).

**Rationale:**ICI-induced DM typically develops rapidly with profound lack of insulin. There is no evidence that high-dose glucocorticoids rescue beta cell function. High-dose glucocorticoids will also increase serum glucose substantially.

**R 4.3** - We recommend treating ICI-induced persistent DM due to islet destruction with s.c. insulin (++00).

**Rationale:**Islet destruction leads to severely reduced insulin secretion. Thus, standard treatment mode is a multiple insulin injection regimen. Age, overall prognosis, and the patient’s abilities should be taken into account when planning the treatment, and target HbA1c should be individualized. The probability of spontaneous recovery is low. There is no evidence that high-dose glucocorticoids will reverse ICI-induced DM.

**R 4.4** - We do not recommend routine complication screening in the first years after the development of ICI-induced DM, taking into account age and prognosis.

**Rationale:**Since ICI-induced DM develops rapidly, we can assume that there are no long-term complications at diagnosis, but we suggest that patients are entered into a screening program. If type 2 DM is suspected, we suggest to include the patient in the regular screening program for complications.

**R 4.5** - We recommend that patients with DM are provided with appropriate and regular training on managing their condition and treatment, including diet and physical activity (Good clinical practice).

**Rationale**: The patients need the same education in self-management as patients with type 1 DM.

### Adrenals

Primary adrenal insufficiency is a rare complication of ICIs, and to date, only few cases have been presented in the literature, making an estimate of incidence very difficult (4, 94). A recent survey of the WHO VigiBase indicated that out of 50 000 ICI-associated adverse events reported since 2008, there were 451 cases of primary adrenal insufficiency of which 46 were considered definitive (95).

Evidence for an underlying autoimmune association has been documented after the use of PD-1 and PD-L1 inhibitors, including atezolizumab (96), pembrolizumab (97, 98), and nivolumab (99). In some of these cases, 21-hydroxylase autoantibodies were present (96, 97), the autoimmune biomarker associated with regular autoimmune adrenal insufficiency (100). Whether patients harbored autoantibodies against the adrenal cortex before treatment with ICI was not clear, and a subclinical autoimmune reactivity might have been present. Several patients had an HLA-genotype associated with autoimmune endocrinopathies. Many cases displayed other organ-specific autoimmune diseases, most often autoimmune thyroid disease, but sometimes also T1D (101).

#### Diagnostic considerations

Diagnosis of primary adrenal insufficiency follows the Endocrine Society guidelines (48). Often a paired morning cortisol and ACTH will give the diagnosis revealing a low cortisol (often below 100 nmol/L) and an ACTH value of more than two times the upper reference limit. If the test results are equivocal, a standard 250 µg synacthen test can be employed which requires a rise in total cortisol level to over 485 nmol/L after 60 min using liquid chromatography-tandem mass spectrometry (102). The cut-off is assay-dependent and in general, immunoassays give slightly higher readings than assays based on tandem mass spectrometry. Beware that the use of oral estrogen will increase total cortisol levels and can give falsely normal results despite the presence of adrenal insufficiency. We recommend to screen for autoantibodies against 21-hydroxylase autoantibodies, as it is a specific marker for autoimmunity against the adrenal cortex. In the setting of malignant disease, we also recommend a CT scan of the adrenal region to detect any primary tumors or metastases.

#### Evidence synthesis

There are no studies on ICI-induced adrenal insufficiency and its treatment of good quality. One study reported on ten patients with adrenal insufficiency (3.5% of the full cohort); one patient was suspected to be in the early stages of empty sella syndrome, and all other patients were either confirmed or suspected of having secondary adrenal insufficiency (see Supplementary Tables for details). Albeit four patients also showed adrenal metastases on imaging (103). All patients were treated with corticosteroids, but no systematic biochemical evaluation after treatment was presented.

#### Recommendations for treatment and follow-up

**R 5.1** - We recommend against the use of high-dose glucocorticoids for treatment of primary adrenal insufficiency (+000).

**Rationale:**There is no evidence for treatment efficacy and high-dose glucocorticoid pose a risk of inducing secondary adrenal insufficiency.

**R 5.2** - We recommend standard glucocorticoid replacement therapy for patients with proven primary adrenal insufficiency (good clinical practice).

**Rationale:**There are no reported spontaneous recoveries after cessation of ICI. If we assume that ICI-induced adrenal insufficiency is caused by the same mechanisms as regular autoimmune adrenal insufficiency, reversibility is very rare although residual adrenocortical function has been reported (104). We recommend hydrocortisone at a dose of 15–25 mg or cortisone acetate 20–30 mg divided into two or three daily doses. Alternatively, the extended release formulation of hydrocortisone, Plenadren® can be used at a dose of 20 mg once daily (105). Prednisolone at 3–4 mg daily is an alternative, but dexamethasone has no place in replacement therapy as there is a high risk of Cushingoid side-effects and HPA-axis suppression.

**R 5.3** - We recommend standard mineralocorticoid replacement for patients with proven primary adrenal insufficiency (good clinical practice).

**Rationale:**There is no evidence of spontaneous recovery after cessation of ICI therapy. Since destruction of the adrenal cortex causes a lack of aldosterone, replacement therapy with fludrocortisone (0.05–0.15 mg) and a diet without salt restriction is recommended. Hydrocortisone and cortisone acetate have some mineralocorticoid effect, prednisolone less so. Thus, a slightly higher mineralocorticoid dose might be needed (e.g. by 0.05 mg) if the patient is switched to prednisolone compared to hydrocortisone and cortisone acetate.

**R 5.4** - We recommend against stopping glucocorticoid and/or mineralocorticoid replacement abruptly without formally checking adrenal function, even if the ICI was stopped (good clinical practice).

**Rationale:**As there is no evidence of recovery, it is essential that adrenal function is checked before corticosteroids are stopped. We recommend the standard 250 µg synacthen test and test of aldosterone and renin levels.

**R 5.5** - We recommend that patients with primary adrenal insufficiency are provided with appropriate and regular training on managing their glucocorticoid treatment during period of illness (‘Sick Day Rules’) (good clinical practice).

**Rationale**: Adrenal crises can be avoided and/or alleviated by early treatment started either by the patient or a relative who has received education in these conditions. Thus, self-management should include training on stress dosing (‘Sick Day Rules’), self-administration of hydrocortisone injection in the event of an emergency and provision of appropriate resources (e.g. steroid emergency card, identification of bracelet/necklace, care plan outlining the management of adrenal crisis and peri-operative glucocorticoid requirement) to enable accurate communication of their conditions and treatment with other healthcare professionals. Consider referral to a patient support group (if available) for ongoing peer-support.

### Parathyroids

Primary hypoparathyroidism is a rare side-effect of immunotherapy, which has been reported in only six cases in the literature (106, 107, 108, 109, 110, 111). Interestingly, some prospective studies reported a high incidence of hypocalcemia in patients on ICI, but this might have been biased by fluctuations in albumin level and acute kidney insufficiency (112). Sri Nalluru *et al.* indeed recently showed in a series of 178 patients treated with nivolumab, pembrolizumab, or ipilimumab that only one patient was actually presenting with hypocalcemia after correction for albumin (113).

In cases of hypoparathyroidism induced by ICI, all the patients described in the literature presented with symptomatic hypocalcemia with intense fatigue, paresthesia, nausea, and generalized weakness. At diagnosis, serum calcium was found to be very low (between 1.25 and 1.62 mmol/L, normal range 2.20–2.55), with undetectable or low-normal PTH levels. Electrocardiograms showed prolonged corrected QT intervals (between 481 and 493 ms) in the four cases in which it was reported.

Three patients presented with hypoparathyroidism while receiving combination therapy with nivolumab and ipilimumab; two were on pembrolizumab alone and one on nivolumab alone (2 cycles and 15 cycles, respectively). Hypoparathyroidism occurred between 2 and 15 cycles of ICI. Most cases of hypoparathyroidism were irreversible. Interestingly, in one patient, hypocalcemia worsened despite the withdrawal of ICI. Even though the expression of PD-1/PD-L1 or CTLA4 is not known in normal parathyroid tissue, Pan *et al.* reported PD-1 expression in 30% of 28 parathyroid carcinomas and 49% of 63 parathyroid adenomas. This might explain why the three cases of hypoparathyroidism occurred after use of at least one PD-1 ICI (114). There are no data indicating that established hypoparathyroidism is reversible.

The autoimmune nature of ICI-related hypoparathyroidism was based on finding activating calcium-sensing receptor (CaSR) autoantibodies in three cases (106, 107, 108). A specific recognition of functional epitopes on the receptor was also demonstrated (106, 107, 108). In one case, CaSR-antibodies were detected at low titer and then considered non-specific (their stimulating activity was not investigated and functionally inert CaSR-antibodies can be found in healthy subjects) (109). Hypoparathyroidism, also accompanied in this patient by colitis and arthritis, clearly improved during glucocorticoid therapy. Thus, the mechanism is in favor of an immune-mediated inflammation within the parathyroid gland.

Hypercalcemia in patients under ICI has also been reported but seems to be non-PTH-mediated in the context of humoral hypercalcemia of malignancy or PTH-related peptide production. Recently, a case of calcitriol-mediated resistant hypercalcemia was reported in a patient treated with nivolumab (115). At present, there is no evidence of direct or indirect effect of ICI therapy on bone metabolism. Nevertheless, calcium and vitamin D supplements should be given to all patients treated with ICI. In addition, bone anti-resorptive agents should be given to those treated with high-dose glucocorticoids.

#### Diagnostic considerations

When hypocalcemia is detected, magnesium, phosphate, 25-OH-vitamin D, and PTH should be assessed.

#### Evidence

Only isolated case reports are available.

#### Recommendations for treatment

**R 6.1** - We recommend against the use of high-dose glucocorticoids for treatment of hypoparathyroidism in patients under ICI treatment; we recommend to give the same treatment as used in hypoparathyroidism due to other causes (++00).

**Rationale:**The major goal is to correct symptomatic hypocalcemia and avoid short- and long-term complications related to hypoparathyroidism or its standard treatment (calcium supplements and active vitamin D) such as paresthesia, neuropsychiatric disorders, renal calcinosis (116).

**R 6.2** - We recommend against the use of recombinant PTH for hypoparathyroidism.

**Rationale:**There are reports of osteosarcoma in rat models after use of teriparatide (PTH1-34) and full-length PTH (117). Although it is considered safe to use in adults with osteoporosis and for replacement therapy in hypoparathyroidism (118), we suggest not to use recombinant PTH.

### Polyendocrinopathy

Polyendocrinopathy is common following ICI treatment, as is also the case with autoimmune endocrine diseases in general. Overall 15–30% of patients with type 1 DM have another autoimmune disease, most commonly autoimmune thyroid disease (119), and in the case of autoimmune primary adrenal insufficiency, polyendocrinopathy is seen in two-thirds of cases (120, 121). ICI-induced polyendocrinopathy is most commonly observed as the combination of thyroid disease and another endocrinopathy; hypophysitis, type 1 DM, or primary adrenal insufficiency.

#### Recommendations for follow-up

**R 7.1** - We recommend that physicians and patients are made aware of the fact that endocrinopathies cluster and screening for other endocrinopathies should be performed with low threshold, even in cases of mild clinical suspicion (good clinical practice).

**Rationale:** Based on the frequency of polyendocrinopathy in regular organ-specific autoimmune diseases, it is to be expected that similar frequencies can occur in ICI-induced endocrinopathies. This is supported by a number of case reports.

## Patient education, empowerment and engagement

Patients who developed endocrine dysfunction during or after ICI treatment will likely require one or more lifelong hormone replacement therapies. Therefore, it is crucial that patients are provided with adequate training and support to effectively manage their condition and ensure continuity of safe care when they require acute care interventions. Effective self-management can be supported through provision of appropriate education, empowerment, and engagement activities.

Comprehensive patient education is an essential element of toxicity management and high-quality cancer care. Enhanced awareness of the expected and possible adverse events improves coping skills and resilience in patients, leading to treatment adherence and improved health outcomes. For effective management of adverse effects, early recognition and treatment initiation are essential. Occurrence or worsening of new symptoms and signs should be reported immediately. Patients should avoid endocrine self-management without coordination with their multidisciplinary team, including an endocrinologist. Patients must also be informed that adverse events can occur at any time and even after ICI treatment arrest.

Specifically, patients with cortisol deficiency should be educated on how to adjust their glucocorticoid treatment during period of intercurrent illness (‘Sick Day Rules’) and trained on the preparation and administration of glucocorticoid injection in the event of an emergency. They should wear a medical alert bracelet/necklace and carry a steroid alert card to communicate appropriate management in case of an adrenal crisis. We encourage that patients with adrenal insufficiency be referred to a patient support group (if available) for ongoing peer support.

Patients with DM should be provided with appropriate and regular training on managing their diabetes including diet and physical activity and referred to a patient support group (if available) for ongoing peer support.

## Conclusions and future directions

ICIs have significant endocrine side-effects that physicians treating these patients need to know about, since they are relatively frequent, and their management differs from other immune-related adverse events in three key ways: (i) ICI therapy can be continued in most cases, (ii) high-dose glucocorticoids are rarely required for management, and (iii) endocrine deficiency usually persists, necessitating lifelong replacement, except for cases with transient thyroiditis.

There is a surprising lack of reports on endocrinopathies related to ICI therapy in large patient databases which include data on adequately diagnosed ICI-induced endocrinopathies in patients followed up over time. As the range of ICIs is expanding, there is a need to establish registries with long-term follow-up, not only to register side-effects but also the outcome of oncologic and endocrine treatments.

## Supplementary Material

Supplementary information

## Declaration of interest

The authors declare that there is no conflict of interest that could be perceived as prejudicing the impartiality of the research reported.

## Funding

The guideline was sponsored by the European Society of Endocrinology http://dx.doi.org/10.13039/100010382.
