# Supplementary material for: Endocrine-related adverse conditions in patients receiving immune checkpoint inhibition: an ESE clinical practice guideline
Source: Eur J Endocrinol. 2022 Sep 23;187(6):G1–G21. doi: 10.1530/EJE-22-0689 (PMC9641795; doi:10.1530/EJE-22-0689)
Supplement: Supplementary information [file supplementary_material.pdf]

Supplementary information

Endocrine-related adverse conditions in patients receiving immune checkpoint inhibition - an ESE clinical practice guideline

Eystein S. Husebye, Frederik Castinetti, Sherwin Criseno, Giuseppe Curigliano, Brigitte Decallonne, Maria Fleseriu, Claire Higham, *Department of Endocrinology,* Isabella Lupi, Stavroula A. Paschou, Miklos Toth, Monique van der Kooij, Olaf M. Dekkers,

Content

Supplementary Table 1: . Main fields of usage of immune checkpoint inhibitors currently approved by the FDA

Supplementary Table 2: Question for the systematic review on treatment of patients with immune checkpoint inhibitor-induced endocrine side effects

Supplementary table 3: Details of included studies

Supplementary table 4: GRADE evidence table

Supplementary Table 1: . Main fields of usage of immune checkpoint inhibitors currently approved by the FDA

Antibody	Tradename	Breast cancer, triple negative	Cervical cancer	Colorectal cancer	Endometrial cancer	Esophageal sqamous cell cancer	Gastric cancer	Head and neck squamous cancer	Hepatocellular cancer	Hodgkin's lymphoma, classical	Lung cancer, non-small cell	Lung cancer, small cell	NHL, large B cell, primary mediastinal	Melanoma	Merkel cell carcinoma	Mesothelioma, pleural	Renal cell cancer	Skin cancer, squamous cell	Urothelial cancer	MSI-high/dMMR cancers	TMB-H solid tumours
CTLA4-inhibitors																					
ipilimumab	Yervoy													x							
PD-1 inhibitors																					
nivolumab	Opdivo			x		x		x	x	x	x	x		x		x	x	x	x	x	
Pembrolizumab	Keytruda	x	x	x	x	x	x	x	x	x	x	x	x	x	x		x	x	x	x	x
cemiplimab	Libtayo																	x			
PD-L1 inhibitors																					
Atezolizumab	Tecentriq	x							x		x	x		x					x		
durvalumab	Imfinzi										x	x							x		

Abbr: MSI: microsatellite instability, dMMR: deficient mismatch repair, NHL: non-Hodgkin's lymphoma, TMB-H: tumour mutational burden high

Supplementary table 2: Question for the systematic review on treatment of patients with immune checkpoint inhibitor-induced endocrine side effects

**Patients**	Adults with cancer receiving immune checkpoint inhibition who develop endocrine-related adverse events. Immune checkpoint inhibition (FDA approved)
**Endocrine-related adverse events**	Hypophysitis<br>Thyroid disorder (Graves' disease, thyroiditis and hypothyroidism, thyroid ophthalmopathy) Hypothyroidism<br>Primary adrenal insufficiency, Type 1 diabetes mellitus<br>Others (hypoparathyroidism, ACTH-dependent Cushing's syndrome)
**Intervention**	Start of immunosuppressive treatment for aim of treatment of the endocrinopathy<br>Hormone replacement, anti-thyroid drugs<br>Dose modification/holding/discontinuation of immune checkpoint inhibition
**Control**	No intervention<br>Best supportive care
**Outcome**	Recovery from adverse event<br>Partial recovery of pituitary axes<br>Ongoing/permanent organ dysfunction<br>Hospitalization<br>Adverse event-related mortality

Supplementary table 3: Details of included studies

ADRENAL INSUFFICIENCY	
**Author**	Ida et al.
**Year**	2020
**Total number of patients (N)**	282
**Follow-up (months)**	13.6
**Immune checkpoint inhibitor (ICI)**	Anti-PD-1
**Adrenal Insufficiency, N (%)**	10 (3.5%) [1]
**ICI stopped/halted, N (%)**	4 (40%)
**High dose steroid treatment, N (%)**	10 (100%)
**Type/dose high dose steroid treatment**	Corticosteroid replacement therapy in n=6, high dose steroids in n =4
**Spontaneous recovery, N (%)**	No systematic biochemical evaluation<br>50% improvement in Performance Status
**Biochemical recovery after high dose steroid , N (%)**	No systematic biochemical evaluation<br>50% improvement in Performance Status

[1] 4 patients with proven AI, 6 with AI suspicion; central AI in 2/10.

PANCREATIC ISLETS		
**Author**	Gauci et al [1]	Kotwal et al.
**Year**	2018	2019
**Total number of patients (N)**	132	1444
**Follow-up (months)**	24	12.9
**Immune checkpoint inhibitor (ICI)**	Anti-PD-1 ± anti-CTLA-4	Anti-PD-1/anti-CTLA-4
**New insulin dependent diabetes, N (%)**	3 (2.3%)	12 (0.8%)
**Spontaneous recovery, N (%)**	0 (0%)	0 (0%)
**ICI stopped/halted, N (%)**	1 (33%)	Unclear
**High dose steroid treatment, N (%)**	0	0
**Biochemical recovery, N (%)**	0%	0 (0%)

[1] The paper also reports on 14 diabetes cases after ICI treatment from a pharmacovigilance database. These cases were less well characterized. In one patient insulin withdrawal was noted.

	PITUITARY						
**Author**	Albarel et al.	Faje et al.	Garon-Czmil et al.	Kobayashi et al	Lam et al.	Levy et al.	Min et al.
**Year**	2015	2018	2019	2020	2015	2020	2015
**Total number of patients (N)**	131	98	94	174	10	17	187
**Follow-up (months)**	33.6			12.2/14.1		13	14.2
**ICI used**	anti-CTLA/placebo	anti-CTLA	anti-PD/anti-CTLA/both	anti-PD/anti-CTLA	anti-CTLA	anti-PD/anti-PD-L	anti-CTLA
**Endocrinopathy N, (%)**	15 (11.5%)	98 (100%) [1]	94 (100%) [1]	16/174 (9%)	10 (100%) [1]	17 (100%) [1]	25 (13%)
**Corticotroph (N)**	13/15	95/97	85 /94 [2]	16/16	10/10	17/17	22/25
**TSH deficiency (N)**	13/15	76/98	20 /94 [2]		5/10	2/17	22/25
**Gonadotroph (N)**	12/15	58/70			5/10	3/16	15/60
**Spontaneous recovery, N (%)**	0%	Corticotroph 0/29 (0%) TSH deficiency 25/29 (86%) Gonadotroph 13/17 (75%)	0 % [2]	0%	Corticotroph 0/10 (0%) TSH deficiency 1/5 (20%) Gonadotroph 3/5 (60%)	Corticotroph 0/9 (0%) TSH deficiency 0 (0%)	Corticotroph 0/10 (0%) TSH deficiency 6/13 (46%) Gonadotroph 5/8 (63%)
**ICI stopped/halted, N (%)**	13%		63%	0%		0%	24%
**Type/dose high dose steroid treatment**	Not specified	HDS			Not specified	Not specified	Various
**High dose steroid treatment, N (%)**	11/15	69	20	0	6	8	15
**Biochemical recovery, N (%)**	Corticotroph 0 (0%)	Corticotroph 4/68 (6%)	One patient with		Corticotroph 0/6 (0%)	Corticotroph 1 (13%)	Corticotroph 0/12 (0%)

	TSH deficiency 11 (85%) Gonadotroph 10 (83%)	TSH deficiency 29/69 (42%) Gonadotroph 18/41 (44%)	corticotroph deficiency recovered (5%)				TSH deficiency 8/9 (89%) Gonadotroph 2/7 (29%)	

HDS: high-dose steroids, anti-PD: anti-PD-1, anti-CTLA: anti-CTLA-4. [1]series with hypophysitis cases
[2] defined as substitution

	THYROID							
**Author**	Ma et al.	Osorio et al.	Peiró et al.	Iyer et al.	Garon-Czmil et al.	Delivanis et al.	Olsson-Brown et al.	Kotwal et al.
**Year**	2019	2017	2019	2018	2019	2017	2020	2020
**Total number of patients (N)**	163	48	73	657		93	90 / 13	91
**Follow-up (months)**			13.9	17.6		8		10.1
**ICI used**	anti-PD +/- CTLA	anti-PD	anti-PD	anti-PD/anti-CTLA/both	anti-PD/anti-CTLA	anti-PD	anti-PD / anti-PD + anti-CTLA	anti-PD-L1
**New thyroid dysfunction, N (%)**	53 (33%)<br><br>Hypothyroidism<br>11 (21%)<br>Thyrotoxicosis<br>42 (79%)	10 (21%)<br><br>Hypothyroidism<br>4 (40%)<br>Thyrotoxicosis<br>6 (60%)	17 (23%)<br><br>Hypothyroidism<br>10 (58%)<br>Thyrotoxicosis<br>7 (42%)	43 (7%)<br><br>All thyrotoxicosis	110 [2]<br><br>Hypothyroidism<br>(55/110)<br>Thyrotoxicosis<br>(55/110)	13 (14%)<br><br>Hypothyroidism<br>6/13 (46%)<br>Thyrotoxicosis<br>7/13 (54%)	13/90 (14.4%) / 3/13 (23.1%)<br><br>Hypothyroidism<br>4 (25%)<br>Thyrotoxicosis<br>12 (75%)	19 (21%)<br><br>Hypothyroidism<br>14 (74%)<br>Thyrotoxicosis<br>5 (26%)
**Recovery, N (%)**	Hypothyroidism<br>0 (0%)<br>Thyrotoxicosis<br>0 (0%)<br>(40/42 transition to hypothyroidism)	Hypothyroidism<br>0 (0%)<br>Thyrotoxicosis<br>0 (0%)<br>(6/6 transition to hypothyroidism)	Hypothyroidism<br>0 (0%)<br>Thyrotoxicosis<br>2 (29%)<br>(4/7 transition to hypothyroidism)	4 (9%) [3]<br><br>(37/43 transition to hypothyroidism)	Hypothyroidism<br>16/55 (29%)<br>Thyrotoxicosis<br>5/55 (9%)	Hypothyroidism<br>1 (17%)<br>Thyrotoxicosis<br>4 (57%)<br>(3/7 transition to hypothyroidism)	Hypothyroidism<br>0 (0%)<br>Thyrotoxicosis<br>0 (0%)<br>(12/12 transition to hypothyroidism)	Hypothyroidism<br>0 (0%)<br>Thyrotoxicosis<br>2 (40%)<br>(3/5 transition to hypothyroidism)
**High dose steroid treatment, N (%)**	9 [1]		2					
**Type/dose high dose steroid treatment**	[1]		Prednisone 0.8mg/kg					
**Biochemical recovery after high dose steroid , N (%)**	0 (0%)		2 Recovery from thyrotoxicosis					

Anti-PD: anti-PD-1, anti-CTLA: anti-CTLA-4. [1] High-dose steroids according to chemo-protocol [2] series with cases only [3] all after high dose steroids

Supplementary table 4: GRADE evidence table

Quality assessment							No of patients Effect (95% CI)[#]	Quality	Importance
**No of studies**	**Design**	**Risk of bias**	**Inconsistency**	**Indirectness**	**Imprecision**	**Other considerations**			
**ADRENAL INSUFFICIENCY**									
**Spontaneous recovery 1 study**						No systematic biochemical evaluation			
**Recovery after high dose steroids 1 study**						No systematic biochemical evaluation			
**PANCREATIC ISLETS**									
**Spontaneous recovery** 2 studies	Cohort studies	Serious [^]	Not applicable with only two studies	No serious indirectness	Serious imprecision (very low number of events)	Heterogeneity in follow-up	0% [!]	⊕OOO Very LOW	CRITICAL
**PITUITARY**									
**Spontaneous recovery 7 studies**	Cohort studies	Serious[^]	No serious inconsistency	No serious indirectness	Serious imprecision	Heterogeneity in follow-up Heterogeneity in underlying treatments	0% for cortictroph insufficienty	⊕⊕OO LOW	CRITICAL
**Recovery after high dose steroids** 7 studies	Cohort studies	Serious[^]	No serious inconsistency	No serious indirectness	Serious imprecision	Heterogeneity in follow-up Heterogeneity in underlying treatments	Corticotroph deficiency recovered in single patients	⊕0OO VERY LOW	CRITICAL
**THYROID**									
**Spontaneous recovery 8 studies**	Cohort studies	Serious[^]	No serious inconsistency	No serious indirectness	Serious imprecision (low number of events)	Heterogeneity in follow-up Heterogeneity in	0% [&] for hypothyroidism Range 0-57%	⊕⊕OO LOW	CRITICAL

						underlying treatments	for thyrotoxicosis		
**Recovery after high dose steroids 2 studies**	Cohort studies	Serious[^]	Not applicable with only two studies		Very serious imprecision	Heterogeneity in follow-up Heterogeneity in underlying treatments	[*]	⊕OOO LOW	CRITICAL

[#] No formal meta-analysis was performed due to large heterogeneity and very low number of patients
[^] No formal protocol for biochemical endocrinological evaluation; no formal protocol to test for recovery
[!] One patient described with recovery of islet function
[&] one study describes a single recovery
[*] Only very few cases described
